# Medical imagery in Maximus of Tyre’s *Orations*

**DOI:** 10.1017/mdh.2023.23

**Published:** 2023-07

**Authors:** Sophia Xenophontos

**Affiliations:** Department of Philology, Aristotle University of Thessaloniki, 541 24 Thessaloniki, Greece

**Keywords:** Maximus of Tyre, Imagery, Medicine, Philosophy, Rhetoric

## Abstract

Imagery is an overarching feature of Maximus of Tyre’s *Orations* which has never been the subject of systematic investigation. This paper provides a starting point by focusing exclusively on medical imagery, one of the most pervasive and instrumental types of imagery in Maximus’ work that has gone entirely unnoticed in the literature to date. This paper shows that Maximus uses medicine (especially its scientific basis and historical development), the physician (e.g. his skill, provision and sensitivity towards the patient), the body (its physiology and workings) and notions of health and disease with considerable diversity and creativity, in ways that make his examples stand out in relation to earlier (Platonic) or contemporary applications of the medical parallel. It argues that the use of the medical imagery in the pedagogical context in which Maximus’ *Orations* were performed facilitated not just clarity but also concept formation and the shaping of a moral outlook as well as the familiarisation with the proper literary references and verbal and conceptual *topoi* for admission into the group of the educated elite. Another main thesis is that medical imagery valorises Maximus’ philosophical status and his claims to Imperial-period acculturation, thus functioning as a trademark for the rhetorical philosophy he wished to promote.

## I

The forty-one lectures that make up Maximus of Tyre’s *Orations* (*Dialexeis*) are an important yet little-studied source for the cultural ambience of late second-century Rome.^1^ With their informal and accessible character, extensive inspiration from classical themes and ideas,^2^ and tendency to address chiefly ethical topics,^3^ the *Orations* offer a fascinating window into the dynamics of philosophical rhetoric as a pedagogical practice in this age. They also cast light on Maximus’ role as a philosophising orator and especially on his moral-didactic approach, given that the professed aim of his *Orations* is to ‘rouse young men’s souls and guide their ambitions’,^4^ as the programmatic first lecture makes clear.^5^

A powerful tool that Maximus deploys to that end is imagery in the broadest of senses: namely, not just the more familiar figures of speech, such as metaphors or similes, but also looser mechanisms of comparison, such as allegories, prosopopoeiae (personifications) and even fables.^6^ An examination of the complete range of forms and functions of imagery in Maximus awaits a comprehensive study.^7^ This paper provides a starting point by focusing exclusively on medical imagery, one of the most pervasive and instrumental types of imagery in Maximus’ work^8^ that has gone entirely unnoticed in the literature to date. This paper will show that Maximus uses medicine, the physician, the body and notions of health and disease with considerable diversity and creativity, in ways that make his examples stand out in relation to earlier or contemporary applications of the medical parallel. By the end of this discussion, I hope that the resulting demonstration of Maximus’ resourceful engagement with medical imagery will substantiate his role as an effective and inspiring communicator and, to some extent, also help further refute modern accusations that he was a second-rate thinker (*Halbphilosoph*)^9^ by foregrounding the idiosyncratic poetics of his orations. On another level, one of the most important contributions of this paper is that it illustrates medicine’s impact on those who were not *stricto sensu* medical writers or medical experts. Thus, it tackles the wider role of ancient medicine beyond the more usual territories explored by the majority of modern scholars. Medicine in the Roman Imperial period (roughly the first three centuries AD) was not just a technical field, but also a vibrant area of study and intellectual engagement, which attracted the attention not just of medical authors and practising physicians but also of other educated elite men, such as sophists and philosophers. This study stresses precisely this less-explored area in the dynamics of ancient medicine as an intellectual field. What is extremely interesting in Maximus’ case is that medicine seems to have interested him a lot as an educational tool, a vehicle for his self-promotion and an innovative means of advertising his public role as a professional orator. This is an aspect of ancient medicine that deserves further exploration, attesting, as it does, to the social role of medicine in Graeco-Roman antiquity.

Before embarking on the main part of the analysis, it seems relevant to mention that Maximus stresses many times throughout the *Orations*, almost in a formulaic fashion, that his use of imagery specifically serves the purpose of clarification and illumination,^10^ just as his use of analogy or mythological and historical parallelism does on other occasions^11^: for example: ‘If an image is needed to illustrate the point’^12^ (εἰ δέ τοι δεῖ καὶ εἰκόνος τῷ λόγῳ); ‘Or if you need a still clearer image, you could imagine God as a general’^13^ (εἰ δέ σοι καὶ σαφεστέρας εἰκόνος δεῖ, νόει μοι στρατηγὸν μὲν τὸν θεόν); ‘Perhaps I need to summon up some other images to illustrate my present theme’^14^ (Εἰ δὲ δεῖ καὶ ἄλλας παρακαλέσαι εἰκόνας τῷ παρόντι λόγῳ); ‘for I realize I am drawing a subtle distinction here and need to give you an illustration’^15^ (αἰσθ<άν>ομαι γάρ τοι ἐμαυτοῦ γλίσχρως τὸ πρᾶγμα διελομένου καὶ δεομένου εἰκόνος) and ‘If you need a still clearer image, this defective state of the soul can be compared to a species of mob rule’^16^ (Εἰ δέ σοι καὶ σαφεστέρας εἰκόνος δεῖ, ὀχλοκρατίᾳ τινὶ εἰκαστέον τὴν τῆς ψυχῆς πονηρίαν).^17^

Taken together, these examples point to the overarching features of Maximus’ image-making in general. First, they show that the employment of imagery is a necessary precondition for driving home any given point Maximus is making in his argument (note the use of the verb δεῖ [*dei*, ‘need’] in almost all cases), and hence an essential factor for his performative and communicative success when addressing young upper-class listeners who sought to be acculturated as much as entertained by his rhetorical skill.^18^ Secondly, the examples also show that the imagery is expected to facilitate concept formation and production of meaning in the young audience, given that an ‘image/illustration’ (εἰκών, *eikon*) is always connected either with the effectiveness of Maximus’ educative discourse or with the specific cognitive abilities that it is expected to foster in them, notably that of imagination (νόει, *noei*, ‘imagine’) and comparison (εἰκαστέον, *eikasteon*, ‘can be compared’).^19^ All the above suggests that Maximus’ employment of imagery is both conscious and tailored to the efficient teacher-pupil interaction that he sets up as a vehicle to enable their attainment of philosophical virtue and successful induction into elite *paideia* (the ideal of intellectual achievement and excellence). In that sense, imagery may be seen as an authority-conferring move on Maximus’ part, especially considering that his notion of the function of the εἰκών as argued above is a modification of the way it is used in his favourite philosopher Plato,^20^ one of the most influential voices in the philosophical landscape of the Roman Imperial period, particularly in the context of Middle Platonism in which Maximus lived and operated.^21^ These points will crop up again as the discussion of medical imagery proceeds.

## II

A ubiquitous theme in the ethical writings of the Hellenistic and Roman periods is the theorisation of moral passions as diseases of the soul (νοσήματα τῆς ψυχῆς, *nosemata tes psyches*), and hence the symbolic representation of philosophy as psychic therapy (θεραπεία τῆς ψυχῆς, *therapeia tes psyches*) targeted at the extirpation of false beliefs and the control of violent emotions through the mediation of philosophical arguments and training.^22^ This therapeutic analogy is evident especially in the Stoic philosophers Epictetus (1st and early 2nd century AD),^23^ Musonius Rufus (1st century AD), ^24^ and Seneca (5 BC–65 AD),^25^ but also beyond strictly Stoic territory, in thinkers such as the middle Platonist Plutarch (circa 45–circa 120 AD),^26^ the Epicurean Philodemus (circa 110–circa 30 BCE)^27^ and others. As a scholar conversant with the philosophical currents of the Second Sophistic (the renaissance of Greek letters from roughly the first to the third century AD), Maximus is aware of the traditional parallelism between medicine and philosophy, which he is already using in his first oration, in the context of a discussion which argues that, unlike the instability of fate, philosophical reason (λόγος, *logos*) is the only steady aspect in human life:

Set over life, however, is Reason (ὁ λόγος), which constantly adapts itself to the circumstances of the moment, like a skilled doctor whose duty is to regulate the indigence and satiety of a body that is not stable, but surges back and forth, in the turmoil of evacuation and repletion (ὥσπερ ἰατροῦ τέχνη ἐπὶ σώματι οὐχ ἑστῶτι, ἀλλὰ φερομένῳ ἄνω καὶ κάτω καὶ ὑπὸ κενώσεως καὶ πλησμονῆς κυκωμένῳ, οἰκονομοῦσα αὐτοῦ τὴν ἔνδειαν καὶ τὸν κόρον). This is precisely what the rational teaching of philosophers can do for human life, adapting its tone to suit the emotions of the moment, so as both to offer consolation in sad times and to enhance the celebrations in times of joy (ξυναρμοζόμενος τοῖς πάθεσιν καὶ πεπαίνων μὲν τὰ σκυθρωπά, συνευφημῶν δὲ τοῖς φαιδροτέροις).^28^

The point Maximus wishes to get across here is that philosophy shares with medicine two positive elements: a) its adaptability to individual circumstances, picking up on the preceding mention of καιρός (*kairos*, ‘intervention at the right moment’)^29^ which is central to ancient medicine,^30^ and b) the preservation of harmony as a guarantor of health. It is interesting that in the quoted passage Maximus does not speak of sickness (νόσος, *nosos*) *as per* the conventional analogy but instead stresses the lack of equilibrium in an unstable body (σώματι οὐχ ἑστῶτι)^31^ that constantly veers between extremes (fullness and emptiness), so as to highlight, by implication, that, in the case of the soul, philosophy is the only possible source of equilibrium.^32^ On the one hand, this choice of presentation serves Maximus’ perennial emphasis throughout his discourses on the notion of symmetry (συμμετρία, *symmetria*), or internal economy, which he considers the ultimate goal of philosophy.^33^ On the other hand, speaking specifically of inconstancy (rather than directly of sickness) may also be explained by the strategic objectives of Maximus’ philosophical display: wishing to imprint the concept even more firmly on his listeners’ minds, the Tyrian associates bodily imbalance with another graphic image, that of rivers, which also represent the fluctuating and the variable. In fact, the dynamic nexus of the unsettled body and the volatile river appears two more times in similar protreptic contexts, suggesting that it was a set formula in the extempore toolkit Maximus deployed to effect his philosophical didacticism.^34^ In adjusting the well-known medical analogy, therefore, Maximus departs from its typical focus on a painful illness that needs to be cured through equally painful means^35^ and recasts it as a positive experience for philosophical beginners by filtering it through an optimistic lens: the type of philosophy opened up to them does not seek to heal a negative aspect of someone in a one-off critical situation but to bring about their emotional well-being in the long run.^36^ This conclusion is arrived at by also considering Maximus’ specific wording in the passage under analysis, where he opts for *oikonomia* (οἰκονομία) rather than of *therapeia* (θεραπεία), so as to advertise the more reassuring aspects of philosophy, that is a provision for maintaining internal balance at all times and not simply restoring it when things go wrong.^37^

It is only fair to note that, even though our author does not refer to an afflicted body in the medical component of his analogy (at least not in any direct sense), when it comes to the philosophical component of the analogy at the end of the quoted section, he gives a more balanced presentation of the conditions liable to affect his pupils in real life by referring to both sadness and happiness. This is no concession, however, because the typical analogy would have dwelt to a greater extent (if not exclusively) on the former (see the examples in note 35). So it is clear that Maximus strives to mitigate the negativity of the stereotypical therapeutic imagery. This is attuned to the kind of philosophy that is on offer here: this is no ‘hardcore’, that is doctrinal or scholastic, philosophy of the sort pursued by Plutarch, Numenius (mid 2nd century AD) or Alcinous (2nd century AD), but a ‘soft’,^38^ ‘undemanding’^39^ variety that imparted philosophical lessons in an encouraging and far from off-putting fashion.^40^ This proposition is supported also by the fact that the adjustment of the imagery to some extent involves shifting the traditional focus from the diseased soul to life itself, with Maximus emphasising the benefits of being able to access the appropriate philosophical discourse in a whole range of life situations (‘sad times … times of joy’) and bringing out the need to have appropriately versatile philosophical discoursers (like him) on tap to produce the right instruction for the right circumstances. The simile, therefore, enables Maximus to present more inspiringly not just philosophy’s status as medicine for the soul but also the performativity of philosophy.

The popularising character of Maximus’ philosophy^41^ is even more evident at the level of language. The medical vocabulary used in the formation of medical imagery is never technical or highly specialised,^42^ while some main tenets regarding the workings of the human body and the physician’s contributions are treated as terra cognita for Maximus’ audience, as they would have been acquainted with the basics of medicine from their so-called ‘circular’ or general studies (ἐγκύκλιος παιδεία, *enkyklios paideia*) and wider cultural background.^43^ A case in point is found in *Oration* 27.3, which provides a definition of physical health as the state in which the blendings of the body’s constituents are harmonised to produce the proportions conducive to health (τὸ δὲ σῶμα ἁρμονίαις καὶ κράσεσιν εἰς ὑγιείας μέτρον).^44^ Ancient scientific thought in Maximus’ day, especially Galenic medicine, which dominated medical theory at the time, referred to health as εὐκρασία (*eukrasia*), a state of good mixture predicated on balancing the four elementary qualities of hot, cold, dry and wet.^45^ As we have just seen, however, Maximus simply describes physical health in general terms, as a harmonious blending, without any specific reference to qualities (ποιότηται, *poiotetai*), which are indeed never mentioned as such in the *Orations* (of which more below), even though the author seems aware of them.^46^ The only detail that Maximus concedes as regards the definition of the body’s constitution is that it consists of cosmic elements (fire, earth, air, water),^47^ a thesis that goes back to Presocratic metaphysics and early Greek medico-philosophical thought including Plato and which was widespread in Maximus’ time (I shall return to this too below).^48^ Leaving that aside, in *Oration* 27.3, too, the use of the medical analogy is straightforward and positive through an emphasis on the bodily and psychic symmetry that Maximus’ philosophy can achieve for his audience.^49^ Hence, the simplification of medical knowledge once more fits in with Maximus’ simple and supportive philosophy.^50^

Another issue meriting attention in this connection is that, in talking about elements in the body, Maximus seems to be drawing specifically on Plato (e.g. *Timaeus* 81e–82a)^51^ rather than being influenced by other classical sources or Imperial-period medical discourse. This is a standard attribute of his deployment of medical imagery, namely, to tap into Platonic references to or invocations of medicine and adjust them to the specific goals and preoccupations of his philosophical orations. In a section that negotiates the topic of learning and recollection with an eye to Plato’s conceptualisation of the phenomenon (viz. knowledge is inherent in us), Maximus relates the following:

Should we call it ‘learning’ (μάθησιν), or should we adopt Plato’s terminology and call it ‘recollection’ (ἀνάμνησιν)? Or should we use both names, ‘learning’ and ‘recollection’, of the one phenomenon? Whatever the answer, the phenomenon itself resembles what can happen to the eye (τὸ δέ ἐστιν τοιοῦτον οἷον τὸ περὶ τὸν ὀφθαλμὸν πάθος). The eye never ceases to possess the faculty of sight, but from time to time some mischance allows a mist to cover and embrace the organ and so block off its contact with the outside world (ἤδη δέ που ὑπὸ συμφορᾶς ἐπιχυθεῖσα ἀχλὺς καὶ ἀμφιέσασα τὸ ὄργανον διετείχισεν αὐτοῦ τὴν πρὸς τὰ ὁρώμενα ὁμιλίαν). When medical science comes to the rescue, its task is not to implant sight in the eye, but rather to remove the blockage so as to uncover it and restore its outward passage (ἡ δὲ τέχνη παρελθοῦσα ὄψιν μὲν οὐκ ἐνεποίησε τῷ ὀφθαλμῷ, τὸ δὲ ἐνοχλοῦν παραναγαγοῦσα ἀπεκάλυψεν αὐτοῦ τὸν ἔξω δρόμον). You must understand that the soul too has a kind of sight, the natural function of which is to discern and understand reality. The misfortune of physical embodiment covers it over with a thick mist (ὑποκεχύσθαι αὐτῇ πολλὴν ἀχλύν), which confounds its powers of vision, removes its precise discernment, and quenches its native brightness. Reason, coming to the soul like a doctor, does not bring and implant understanding (λόγον ὥσπερ ἰατρὸν οὐ προστιθέναι αὐτῇ φέροντα ἐπιστήμην), like something the soul did not already possess; instead, it reawakens the understanding it does possess, but which is dim and constrained and torpid.^52^

That Maximus had taken his inspiration from Plato’s *Republic* 518d for the imagery relating to the eye could scarcely have been missed by his audience, who were well versed in the Platonic tradition. Plato’s point is merely that there is an art (philosophy) which effectively reawakens the soul so as to enable it to ‘see’ (conceive, understand), but it does not produce sight in the eye as if the capacity for vision was not inherent in it already. Maximus’ reworking of the eye imagery is both clearer and more developed: a) it overtly introduces medicine (absent from Plato) as a parallel system to philosophy, b) it elaborates on the blocking of vision through ‘mist’ (ἀχλύν, *achlyn*), eliciting an imaginative involvement on the part of the audience, as this would have been an uncomfortable eye condition that they would have experienced either in themselves or vicariously^53^ and c) it corroborates the idea that the soul has innate knowledge by combining the borrowing from Plato’s *Republic* 518d, as seen above, with another medically related imagery, that of the art of midwifery derived from *Theaetetus* 149a–151d (cf. *Phaedrus* 276b–277a) which is instrumental in showing that ‘reason plays midwife to the pregnant soul’ (*Oration* 10.4),^54^ exactly in line with what is suggested in the passage quoted above. Of course, a further attraction of the parallel with midwifery is that it turns the spotlight onto Socrates and his salient role in philosophical education, as advocated by Maximus.

Indeed, Maximus is very keen on employing medical imagery when he wants to reinforce Socrates’ (circa 470–399 BC) philosophical authority and thereby capitalise on the principles from Socratic/Platonic philosophy that he expects his auditors to assimilate. For example, in *Oration* 3.1, in order to underline how paradoxical the denigration of Socrates has been on the part of successive generations of his detractors up to Maximus’ own day, the latter presents Socrates as an expert in matters philosophical, who nevertheless did not enjoy the same respect as other specialists, one of whom is the physician:

What a monstrous discrepancy! Of the other crafts and sciences, each and every one is free from the jurisdiction of the multitude. The steersman, when he takes command of his ship and exercises his science according to its own proper principles, is not held to account by laymen; the doctor does not have to put up with his patients reviewing and examining his recommendations and the cures and regimes he suggests (μήτε τὸν ἰατρὸν ἀνέχεσθαι τοὺς κάμνοντας τὰ προστάγματα αὐτοῦ καὶ τὰ ἰάματα καὶ τὰ διαιτήματα ἐπισκοποῦντας καὶ βασανίζοντας); nor are potters or leatherworkers, or the practitioners of still more lowly pursuits than these, answerable to any other judge of their activities than their own craft. But Socrates, whom not even Apollo–he who knows the numbers of the sands and can divine the measures of the sea–could convict of ignorance, has not ceased to this very day to be the object of accusation and investigation.^55^

The superior wisdom and expertise of the physician as well as that of the steersman and of other professionals (the painter and the sculptor are also brought into the discussion later on) are enough to shield them from the criticism of laymen and ensure them general approbation. So why is it not counterintuitive when Socrates, whose proficiency was not some manual skill, but something more elevated involving the symmetry of life (ἀλλὰ τὸν αὑτοῦ βίον συμμέτρως), is not appreciated? This is the question that Maximus problematises for his audience and which he accentuates through the additional emphasis on Socrates’ virtues, notably his simple lifestyle (εὐτελείᾳ), perseverance (καρτερίᾳ) and self-control (σωφροσύνῃ), encouraging his listeners to be inspired by Socrates’ moral grandeur.

Examples such as this could be multiplied (e.g. *Oration* 8.3), and the use of medical analogies for didactic purposes becomes even more intriguing. The following case study shows that, in order to highlight Socrates’ powers of persuasion, Maximus includes examples from other authorities who have, in fact, been eclipsed by Socrates’ persuasive talents:

What doctor ever persuaded his feverish patients that going without food and drink was a good thing? (ἢ τίς πώποτε ἰατρὸς ἔπεισεν τοὺς πυρέττοντας ὅτι ἀγαθὸν τὸ διψῆν καὶ λιμώττειν;) Who ever persuaded a hedonist that his aims were worthless? Who ever persuaded the money-grubber that his object is not a good? To be sure, Socrates would have had no trouble at all in persuading the Athenians that the pursuit of Virtue is not the same thing as corrupting the young, and that knowledge of the divine is not the same thing as irregularity in religious observance.^56^

Unlike the image of the hedonist and the money-grubber, the one with the doctor involves an audience to whom the latter directs his persuasive competence, making it a better match to Socrates’ relationship with his accusers. The image of the unpersuasive doctor (like that of the money-grubber) originates in the *Gorgias*. However, in Maximus’ hands, it is transformed on two levels. First, Maximus is inspired by the famous section 456b ff., where Gorgias explains how he, as an orator, was able to persuade the patients of his brother, the physician Herodicus, to accept drugs, surgical operations or cauterisation in instances where the latter was unable to do so. Maximus supplements the element of persuasion that dominates this section from the *Gorgias* with references to food and drink that occur in another famous passage from this dialogue,^57^ the one staging the confrontation between a doctor and a cook, resulting in the former’s crude victimisation by the latter. Thus, in constructing his own medical imagery, Maximus makes an ingenious fusion of two Platonic parallels, as already seen in the case of the eye imagery above. Second, he simultaneously adds his individual ingredients, in this case, the fact that the patients refusing food and drink suffer from fever, an image used in exactly the same notional and linguistic form in *Oration* 25.5. This suggests that this was a set image, easily retrieved in an oral delivery; while focusing specifically on ‘feverish’ patients could spark some recognition in his audience (just like the mist obscuring the eye cited above) and thereby help Maximus cement the image’s impact on them, making its message even more perceptible.^58^

The aspect of pleasure that normally differentiates the cook from the doctor in Platonic settings is another fundamental aspect in Maximus’ shaping of the medical illustration, which is used so as to make or clinch a point in his demonstration. For instance, in order to undermine pleasure as a criterion for defining the good friend (given that the friend’s constructive criticism can be painful), Maximus mentions that ‘it is the benevolent doctor who causes the greatest pain, and the most scrupulous general, and the most reliable helmsman’.^59^ Nonetheless, on another occasion, Maximus refashions this Platonic intertext by assigning the ability to please to the doctor too (i.e. not just the cook) but only when it benefits the patient’s health: in arguing that the use of myth has helped make philosophy more amusing to its audiences, Maximus compares the situation with physicians who, when ‘faced with patients who make problems about taking their medicine, immerse their bitter drugs in pleasant food and thus conceal the unpleasantness of the cure’.^60^ Ways of making his philosophy entertaining and approachable are what concerns Maximus on a metanarrative level, too, with his employment of imagery potentially functioning as a ‘pleasant food’ in which the hard stuff of philosophy is integrated.

The functional adjustment of philosophy^61^ is a major concern for Maximus, and so, in legitimising the use of lies when advantageous to the moral agent, our author again borrows the medical model from Plato^62^ when he says that ‘doctors deceive their patients…and there is nothing very terrible in that. Quite the reverse, indeed: lies have often helped people and the truth has often harmed them’.^63^ Remarkably, when it comes to poetic falsehood and the indulgence that poetry engenders, Maximus is reluctant to take a flexible stance or yield to adjustment. Rather, he vindicates Plato’s decision to expel Homeric poetry from his ideal city on the grounds that the special character of Plato’s state rendered poetry’s utility and entertainment redundant. He does so in medical terms by emphasising that in the framework of Platonic idealism – where everything is well thought out and harmonious – medicine, too, would be unnecessary:

No: Plato’s foundation and his republic are established in purely theoretical terms; he aims for the greatest possible perfection rather than for what might be most practicable–just like those sculptors who bring together beautiful elements from all over, using their art to combine details from many different bodies into one single representation, so as to produce a single, sound, well-constructed, and harmoniously beautiful artefact. You wouldn’t be able to find a real body that exactly resembled such a statue, because art aims at what is most beautiful, while the things we encounter and use in everyday life fall short of what art can produce. I suppose that if human beings had the power to sculpt bodies of flesh and blood, then our craftsmen would be able to mix together, in the right proportions, the quantities of earth and fire (τὰς δυνάμεις ξυμμέτρως γῆς καὶ πυρός) and everything else that when harmonized and co-ordinated with them constitute our bodily nature, and so presumably produce a body that had no need of drugs and quack remedies and the regimens of doctors (ὡς τὸ εἰκὸς ἂν σῶμα ἀδεὲς φαρμάκου καὶ μαγγανευμάτων καὶ διαιτημάτων ἰατρικῶν). Suppose then that someone heard one of those craftsmen legislating for his theoretical creations and saying that they had no need even of a Hippocrates to heal them (δεήσονται οὐδὲ Ἱπποκράτους ἰωμένου σφᾶς), but that they ought to crown the man with wool and anoint him with myrrh and send him somewhere else, to win his reputation where sickness made his arts necessary (εὐδοκιμήσοντα ἐκεῖ ὅπου τὴν τέχνην παρακαλεῖ ἡ νόσος); and suppose that our hearer grew angry with the craftsman for dishonouring the art of Asclepius and the Asclepiadae (ὡς ἀτιμάζοντα τὴν Ἀσκληπιοῦ καὶ τὴν Ἀσκληπιάδων τέχνην). Wouldn’t he be making a laughing-stock of himself by bringing an accusation against someone who was not rejecting medicine because he scorned it, but because he neither needed it for practical purposes nor welcomed it as a source of pleasure (ἆρα οὐ καταγέλαστος ἂν γίγνοιτο, αἰτίαν προσφέρων τῷ μὴ κατὰ ἀτιμίαν παραιτουμένῳ ἰατρικήν, ἀλλὰ μήτε κατὰ χρείαν δεομένῳ αὐτῆς μήτε καθ’ ἡδονὴν ἀσπαζομένῳ)?^64^

Even though this section is heavily informed by Plato’s *Republic*, both in terms of quotation and allusion,^65^ the final product is a creative synthesis that is Maximus’ own. The presentation of the unified, harmonious body consisting of proportionate elements and the mention of drugs, remedies and medical regimens being unnecessary interventions in a well-balanced body are used elsewhere by Maximus^66^ to underpin his idea of physical and (by comparison) mental harmony. In addition, the device that has the addressee confront the provocative scenario in which medicine is dismissed as an impractical science but with good reason is a decisive move towards reinforcing the independence of Plato’s theoretical construct from any human art, including medicine. Interestingly, in the last sentence of the quoted section, alongside presenting medicine as a hands-on science (a common attribute of the medical art), Maximus also portrays it as a source of pleasure. What exactly our author has in mind is hard to tell,^67^ but what matters for our purposes is that the pleasurable component of medicine is a variable that Maximus shifts depending on the nature and needs of his exposition in any given instance. Here, it makes sense to assert the pleasurable aspect of medicine (despite the attribute’s conflicting connotations) in conjunction with its practicability to make it precisely analogous to poetry’s own practicability-cum-pleasure. That said, in the majority of occurrences, Maximus is intent on portraying pleasure as inimical to medicine, drawing on the Platonic antithesis between medicine and cookery:

…just like fever patients who gorge themselves on food and drink against doctor’s orders (ὥσπερ οἱ πυρέττοντες, ἐμπιπλάμενοι ποτοῦ καὶ σιτίων παρὰ τοὺς τῆς τέχνης νόμους). Comparing one evil (disease) (νόσῳ) with another (exertion) (πόνους), they prefer to be sick and enjoy themselves (αἱροῦνται ἡδόμενοι νοσεῖν), sooner than to exert themselves and be cured (μᾶλλον ἢ πονοῦντες ὑγιασθῆναι). Many a resourceful doctor has before now tempered the bitterness of his cure with a small admixture of something sweeter; but neither Asclepius nor the Asclepiadae are indiscriminate purveyors of pleasure–that is the work of caterers (καί τις ἤδη ἰατρὸς εὐμήχανος ἀνεκέρασεν βραχεῖαν ἡδονὴν τῷ ἀλγεινῷ τῆς ἰάσεως· ποριστὴς δὲ ἡδονῆς καὶ παντοίας ἡδονῆς οὔτε ὁ Ἀσκληπιὸς οὔτε οἱ Ἀσκληπιάδαι, ἀλλ’ ὀψοποιῶν τὸ ἔργον).^68^

The juxtaposition of medicine and cookery in Plato is a motif which Maximus frequently deploys to develop with more clarity the archetypal distinction between crafts (τέχναι, *technai*) and ‘knacks’ (ἐμπειρίαι, *empeiriai*): crafts, based on accurate knowledge of a subject, benefit the soul or body, like medicine which cares for the body in a genuine sense. Knacks, by contrast, based on mere imitation of crafts, seek to flatter the audience regardless of their well-being. The knack that imitates medicine is cookery, targeted at pleasing the body, thereby engendering its destruction. Maximus seems well acquainted with these and other related insights.^69^ This is attested, for example, by his treatment of the story of Mithaecus, a celebrated chef who was expelled from Sparta accused of overfeeding the ‘unpampered and pure’ bodies of the Spartans and thus going against their simple, nourishing diet.^70^ The fine line between medicine and cookery is clearer in the following comparison, which is employed by Maximus to explain that sophistry is the flattering variety of philosophy:

Men were exposed to the flattery of a bogus form of medicine, when they abandoned the healing techniques of Asclepius and the Asclepiadae and reduced science to something indistinguishable from gourmet cookery, a substandard flatterer to substandard physiques (ἐκολάκευσεν ἀνθρώπους καὶ ἰατρικὴ νόθος, ὅτε τὴν Ἀσκληπιοῦ καὶ τὴν Ἀσκληπιαδῶν ἴασιν καταλιπόντες οὐδὲν διαφέρουσαν ἀπέφηναν τὴν τέχνην ὀψοποιϊκῆς, πονηρὰν κόλακα πονηρῶν σωμάτων). The informer imitates the orator, setting argument against argument, fortifying injustice against Justice and the base against the noble. The sophist imitates the philosopher. He is the most scrupulous imitator of them all.^71^

The reference to Asclepius and the Asclepiadae^72^ is Maximus’ own addition to *Gorgias* 464d, which seems to be distantly informing the above passage, and it is there to achieve a two-fold aim. First of all, it ensures that the comparison makes a greater impression on Maximus’ listeners by spelling out the name of the healing god and his priests. At the same time, it renders the contrast between real crafts and their imitative versions more successful by emphasising that medicine had been brought down to the level of cookery through the relinquishing of the therapeutic practices of Asclepius himself, who is portrayed as the exponent *par excellence* of the summit of medicine throughout Maximus’ *Orations.* ^73^ That Asclepius is so dear to Maximus’ heart that he uses him at strategic points in his demonstration may be explained partly by his reliance on Plato^74^ and partly by the fact that in the Imperial era, Asclepius as a god was held in widespread affection.^75^

Turning now in more detail to the image of the body and its diseases, it could be said that Maximus makes use of it chiefly in order to clarify the meaning of abstract and complex philosophical notions. To better explain that pleasure has its basis in the enjoyment of the experiencing subject, not in its own nature, he compares it with the nourishment of the body, which works exactly the other way around.^76^ The reference here to the operation of the digestive system, which again does not involve anything beyond the commonsensical,^77^ is effectively used as a form of argumentative elucidation. Elsewhere, in order to show that love needs reason (rather than emotion) to be rendered a virtue and not a sickness, Maximus employs a parallel from health, which likewise comes about not through emotion but through nature or human artifice:

If Love is an impulse towards friendship, and a desire of one like thing speeding naturally to meet its like and straining to combine with it (which would be a phenomenon of emotion, not of reason), then the supervision of reason will have to be added to this emotion in order to make of it a virtue rather than a sickness. Just as in the case of our bodily constitution, health is a certain non-rational condition of the forces of wetness, dryness, cold, and heat, neatly blended by human artifice and artfully harmonized by Nature–and if you remove anything of the contribution made by Nature or by artifice, you will have upset this non-rational state and driven health away (καθάπερ γὰρ ἐπὶ τῆς τῶν σωμάτων κράσεως καὶ ἡ ὑγεία πάθος τί ἐστιν ὑγρῶν καὶ ξηρῶν καὶ ψυχρῶν καὶ θερμῶν δυνάμεων, ἢ ὑπὸ τέχνης συγκραθεισῶν καλῶς ἢ ὑπὸ φύσεως ἁρμοσθεισῶν τεχνικῶς, ἂν δὲ ἀφέλῃς τῆς φύσεως ἢ τῆς τέχνης, τὸ μὲν πάθος συνετάραξας, τὴν δὲ ὑγείαν ἐξήλασας)–so it is in the case of Love: even if it enjoys the control of reason it remains an emotional state, and if you remove reason, you will have disturbed its equilibrium and converted it wholesale into sickness (ἐὰν δὲ ἀφέλῃς τὸν λόγον, ἐπετάραξας αὐτοῦ τὴν συμμετρίαν καὶ νόσον ἐποίησας τὸ πᾶν).^78^

The comparison between bodily health and love that Maximus proposes here is most probably influenced by the connection between bodily health and justice in the soul from *Republic* 444c–d. Nevertheless, nowhere in this passage does Plato advance the definition of somatic health as a non-rational condition (πάθος, *pathos*) of the forces (δυνάμεις, *dynameis*) of hot, cold, dry and wet, blended by means of the medical art and harmonised by nature; Plato simply speaks of bodily balance in a general sense. What is more, even though Maximus refers here to hot, cold, dry and wet, he does not label them as ‘qualities’ but simply as ‘forces’, a concept that would have made perfect sense to his recipients. By the same token, in other cases, nouns referring to the constitution of the body remain unqualified by Maximus, notably through his use of substantive adjectives accompanied by the definite article: for example, τἀναντία for ‘opposing elements’.^79^ This shows that in the framework of his unchallenging philosophy, Maximus gives little – if any – attention to the niceties of scientific theory, paying more heed to make his phrasing readily intelligible to his young audience. Baffling the latter with a scientific articulation of medical terms and ideas at any point in the lecture would have meant Maximus’ outright failure, putting at risk the entire enterprise of his pedagogical project. The same can be said of Maximus’ flexible use of terminology that would have otherwise been used in a very specialised sense in the context of technical treatises of the time. For example, κρᾶσις (*krasis*) in Maximus never denotes ‘mixture of elements’ but rather points more generally to the body’s constitution, attesting to an intuitive appreciation of the body’s organic unity on the audience’s part, just like δύναμις (*dynamis*) above could hardly have been construed in its specialised meaning of ‘capacity’, but rather as referring more generally to ‘power’, as noted. Maximus could simply not afford to be obscure in oral performance, and hence any jargon had to be strictly avoided.

The reference to Asclepius mentioned above requires further discussion because the figure of Asclepius is a potent one in Maximus’ rhetorical apparatus. Beyond the fact that Maximus portrays Asclepius as the god of health (δαίμων, *daimon*), responsible for healing the sick,^80^ he also highlights his personal connection with him when he affirms that he has seen Asclepius in waking reality and not just during an incubation in his temple like most ordinary people.^81^ This personal testimony of epiphany experienced by Maximus does not just consolidate the credibility of his account or make it more memorable, as has been suggested,^82^ but notably authorises his discourse, particularly when speaking about medical issues. A central theme of his medically informed discussions in the *Orations* is the knowledge Maximus claims to possess about the invention and history of medicine. As far as its invention goes, Maximus gives a description of how medicine first became a science by means of the accumulated records of the sufferings of patients and the ensuing therapeutic measures that proved effective for the majority of them.^83^ As far as history goes, he is fond of negotiating medicine’s decline (as perceived by Imperial-period pessimistic observers) by comparing it to its earlier sublimity in the distant past. In a passage where Maximus seeks to show that philosophy is a developed form of poetry, he trades on medicine’s history to illuminate the point:

What is philosophy, if not a younger form of poetry, less formal in composition and more lucid in expression? If then these two differ from each other only in age and in superficial form, how ought one to understand the difference in what the two kinds of composer-poet and philosopher-say about the gods? Could we perhaps say that this present enquiry of ours is like someone comparing medicine in its original form with the modern form that treats the patients of today (οἷον εἴ τις καὶ ἰατρικὴν ἐνθυμηθεὶς τὴν πρώτην ἐκείνην πρὸς τὴν νέαν δὴ καὶ τοῖς νῦν σώμασιν ἐπιτεταγμένην), and examining the weak and strong points of each? Asclepius would inform this enquirer that other arts and sciences remain unaffected by the passage of time: where the need remains constant, the response does not vary either. The science of medicine, however, is constrained to adapt to the physical constitutions of its patients, which do not constitute a stable and clearly defined factor, but are modified and changed by the diets that go with different styles of life (ἰατρικὴν δὲ ἀνάγκη ἑπομένην τῇ κράσει τῶν σωμάτων, πράγματι οὐχ ἑστῶτι οὐδὲ ὡμολογημένῳ, ἀλλὰ ταῖς κατὰ τὴν δίαιταν τροφαῖς ἀλλοιουμένῳ καὶ μεταπίπτοντι); different treatments and therapeutic regimes, adapted to the dietary habits of the moment, must be devised for different eras (ἰάματα καὶ διαίτας αὐτῷ ἐξευρίσκειν ἄλλοτε ἄλλας, προσφόρους τῇ παρούσῃ τροφῇ). ‘So do not think’, Asclepius would continue, ‘that my famous sons, Machaon and Podalirius, were any less skilled in the art of healing than those who have set their hands to it subsequently, and thought up their various clever cures. At the time my sons were working, the bodies that their arts had to treat were not degenerate and oversophisticated and completely enervated; as a result they found them easy to cure, and their function was a simple one: *Cutting out arrows, and smearing on soothing drugs* (*Iliad* 11.515). But as time went on the human body slipped out of the control of this archaic form of medicine, fell prey to a more sophisticated style of living, and developed flaws in its constitution (ὑπολισθαινόντων αὐτῇ τῶν σωμάτων εἰς δίαιταν ποικιλωτέραν καὶ κρᾶσιν πονηράν), with the results that we now see: medical science has itself had to become more sophisticated (ἐξεποικίλθη καὶ αὐτή), and to exchange its former simplicity for something more complex.’ Come now, following Asclepius’ example, let the poet and the philosopher together defend their pursuits to us.^84^

Two topics merit consideration here. First, Maximus mentions that, owing to the degeneracy of the human body, medicine became more diversified so as to be able to respond to the treatment of different bodily malfunctions. The refinement of medicine is a negative thing, according to Maximus (despite medical authors’ claims to the contrary) because it militates against its authentic form as a single, unified science.^85^ Taking his cue from Plato, Maximus is an ardent proponent of simplicity and uniformity on all fronts, as mentioned above, a tendency that also informs his conceptualisation of an archaic kind of science, which offered a holistic psychosomatic therapy,^86^ dispensing with the need for disciplinary division in treating the body separately from the soul. This ideal form of science was practised by the centaur Chiron, traditionally considered the inventor of medicine and Asclepius’ teacher in the medical art.^87^ Another reason why Maximus rejects specialisation altogether is because he believes it arises out of the need to combat disease engendered from the disruption of συμμετρία (*symmetria*), the optimum balance in the embodied soul.^88^ For example, in *Oration* 39.2 he expresses dissatisfaction with what he perceives as specialised areas of medicine arising from attempts to treat imbalance *qua* disease.

Secondly, the decline of medicine is a recognisable trope in Imperial-period literature, reflecting the cultural anxieties of this era, particularly the nostalgia for the great Greek past and the aspirations of Second Sophistic authors to invoke and emulate their superior Classical predecessors. Yet, Maximus (again) does not seem to have taken the theme from his contemporary intellectual trends, which, in fact, tackled the issue rather differently: Galen, for instance, also talked about the decadence of medicine in his day but presented it in terms of the failure of medical practice and the moral bankruptcy of medics.^89^ Maximus does not seem to have used Plato^90^ as an independent support either. Rather, it appears that he, in part, invented (or at any rate massaged) the story of the modification of medicine over time to make it an illustrative, supporting parallel to how poetry of the revered past had to change into more explicitly formulated philosophy because human minds became more petty and quibbling over time. As the passage above^91^ makes clear, through the historical development of medicine, Maximus, for his part, associates sophistication with a lack of naivety on a philosophical plane to encourage his audience to adopt simplicity of manners. The mode of presentation, in this case, that is, the prosopopoeia of Asclepius talking about the early form of medicine, makes the narrative appealing, lively and particularly influential.

The same technique features in another instance, where this time, Maximus presents God as caring for the creation as a whole and not for particular aspects, like a sensitive doctor, whose aim is similarly to preserve the entire organism and not its individual parts. The personified Asclepius, this time, castigates the limbs for acting selfishly and disregarding the organism’s status as an integral whole:

Do you want God to care for creation as a whole? Then do not importune him; he will not listen to you if what you ask militates against the preservation of the whole. What would happen if the limbs of the body gained voices, and if when one of them grew tired of being operated on by the doctor in the interests of the body as a whole, they prayed to Medicine not to be destroyed (τί γὰρ εἰ καὶ τὰ μόρια τοῦ σώματος φωνὴν λαβόντα, ἐπειδὰν κάμνῃ τι αὐτῶν ὑπὸ τοῦ ἰατροῦ τεμνόμενον ἐπὶ σωτηρίᾳ τοῦ ὅλου, εὔξαιτο τῇ τέχνῃ μὴ φθαρῆναι)? Would not Asclepius reply to them like this: ‘Miserable creatures, it is not for the whole body to be ruined to serve your interests; it is for you to perish in order that it should be saved’ (ὦ δείλαια, χρὴ οἴχεσθαι τὸ πᾶν σῶμα, ἀλλὰ ἐκεῖνο σωζέσθω ὑμῶν ἀπολλυμένων). Precisely the same is true of creation as a whole. The Athenians suffer from the Plague, the Spartans suffer earthquakes, Thessaly is flooded by tidal waves, and Aetna erupts. You may call such breakings-up ‘destruction’, but the true doctor knows their cause (ὁ δὲ ἰατρὸς οἶδεν τὴν αἰτίαν); he disregards the prayers of the parts and preserves the whole (ἀμελεῖ εὐχομένων τῶν μερῶν, σώζει δὲ τὸ πᾶν), for his concern is for creation at large (φροντίζει γὰρ τοῦ ὅλου). Although God’s Providence does in fact extend to particulars as well. But prayer is out of place there too, being like a patient asking his doctor for food or medicine on his own initiative (ὅμοιον ὡς εἰ καὶ ἰατρὸν ᾔτει ὁ κάμνων φάρμακον ἢ σιτίον): if it is efficacious, the doctor will give it unasked (τοῦτο γὰρ εἰ μὲν ἀνύτει, καὶ μὴ αἰτοῦντι δώσει); if it is dangerous, he will withhold it even when asked (εἰ δὲ ἐπισφαλές, οὐδὲ αἰτοῦντι δώσει). To sum up: nothing that falls under the heading of Providence is to be requested or prayed for.^92^

The idea of individual body parts that should operate in the best interests of the whole organism appears in other authors of this period.^93^ Maximus’ divergences are obvious: he both fabricates the dialogue between Asclepius and the selfish bodily limbs and unpacks the whole–part concept by intertwining it with the image of God *qua* doctor.^94^ Along with his generally Platonising standpoint, Maximus’ relation to Stoic accounts of the body here may be another aspect of his rehabilitation from the pejorative label of the ruminate philosopher.^95^ Moreover, unlike the way in which the treatments mentioned above are used, Maximus dwells on the whole–part concept in another set of instances which illustrate how political order and social justice are attained: for example, just like diseased bodily members have to be cured to halt the damage spreading to the rest of the body, ‘so when a first act of injustice afflicts a household or a city, the canker must be stopped if the remainder is to be saved’.^96^ In particular, *Oration* 15.4–5 presents close similarities with *Oration* 5.4 cited above in that it likens the function of political society to the function of the bodily parts and in that it frames this comparison as a fictional dialogue between the body members and a Phrygian storyteller, who plays a similar role to that of Asclepius in the passage cited above.

## III

All in all, this contribution has attempted to explore the multifarious ways in which Maximus of Tyre adjusts the medical imagery in his rhetorical speeches designed to familiarise his young pupils with the characteristic aspects of philosophy, especially moral philosophy. We have seen that in the pedagogical context of Maximus’ *Orations*, the use of the medical imagery facilitates not just clarity but also concept formation and the shaping of a moral outlook as well as the familiarisation with the ‘right’ literary references and (verbal and conceptual) clichés for admission into the club of the cultivated and the philosophically woke. We have shown that Maximus works with the typical analogy of philosophy as therapy of the soul but adjusts it to the demands of his philosophical teaching, particularly the need to be accessible as well as appealing so as not to discourage the members of the social elite who have just embarked upon their engagement with philosophy at an advanced level. We have also argued that Maximus accentuates various aspects of the medical encounter between doctor and patients (e.g. the recalcitrant patient who refuses to submit himself to the doctor, the doctor’s persuasion of the former, pleasure in medicine), the workings of the human body and the decline of the art of medicine to enable his young listeners to make sense of his argument and be persuaded by his admonitions. Moreover, we have remarked that Maximus uses the physician as an image for God and that he often connects medicine with Asclepius to stress its ideals of balance and stability but also to bestow authority on his own practice. As has been noted, Maximus’ medical imagery makes no use of medical technicalities and does not show any sharp contemporary social detail. What does emerge, however, is an important rhetorical point about what medicine as a given source domain makes available to the teacher/orator and how the skilled teacher/orator makes use of it. Medicine does not provide a fixed set of one-to-one readymade correspondences but rather a field of possibilities that is highly flexible in its application. According to need, different details from the domain can be focused on (physiology, doctor behaviour, patient behaviour, etc), and the same detail can be applied to a range of different targets. In that sense, a lot of Maximus’ skill as an orator/teacher lies in the perceptiveness with which he sees how the resources of the field can be turned to advantage differently in each new moment of need.

That Maximus’ work is full of medical references and associations reflects contemporary trends, given that health talk was part of mainstream elite culture in Maximus’ day, as can be seen from relevant discussions in Plutarch, Gellius (circa 125–after 180 AD), Aurelius’ correspondence with Fronto (circa 100–late 160s AD), and of course Aelius Aristides (117–181 AD). However, it also highlights Maximus’ own knowledge of Greek culture, and more especially, his distinctive ways of translating and appropriating that tradition. Medical imagery, therefore, valorises Maximus’ philosophical status and his claims to Second Sophistic cultural capital, functioning as a trademark for the rhetorical philosophy he wished to parade, which was, to his mind, still respectable, proper philosophy in this period, just as he was himself a major player in the contemporary philosophical tradition (albeit a self-proclaimed one).

Above all, the foregoing analysis has brought out the fact that medical imagery in Maximus does not simply reflect reality so as to make the speaker’s thought and material easier for his audience to digest, but rather it actively shapes and orients the audience’s moral decision-making and associated behaviour. The most important ethical behaviour Maximus sought to encourage in them through the analogy with the medical discipline is the cultivation of moderation, self-control and conscious deliberation. In that sense, the employment of medical imagery in Maximus can be correlated with the function of modern Concept Formation theory, which postulates that metaphor, including imagery, is not a decorative literary device in human culture but a conceptual tool to think and act with.^97^ The same can be said of Maximus’ overall approach to medicine, which despite an emphasis on its scientific basis (e.g. a focus on the prognosticating ability of the physician^98^ or his professional judgment in diagnosis)^99^, is presented as culturally and socially informed in the *Orations* and is thus seen through the lens of medical anthropology which places health and disease at the forefront of human society, never at its periphery as a distant or irrelevant science. As we have seen, the medical metaphor in Maximus draws significantly on the role of medicine as experienced in the world around him and his audience, regardless of its Platonic precedents and invocations which, as we have noted, are meaningfully adjusted to Imperial-period reality. While passively reflecting what the speaker wished to say, medical images in Maximus actively affected the way his listeners thought and behaved, acting at the same time as both mirror and agent.

## Footnotes

1 Biographical details on Maximus are scarce. The three main sources are Eusebius (ca. 260–339 AD), the *Suda* (a Byzantine encyclopaedia of the 10th c. AD) and the manuscripts preserving his *Orations* (e.g. the main witness *Parisinus graecus* 1962), but all are of questionable accuracy. What can be ascertained is that Maximus was active in the reign of Commodus (180–192 AD) and that his lectures were delivered during a stay in Rome. The reconstruction of Maximus’ life is also hampered by the absence of biographical self-reference in the *Orations.* For discussions of his biography and the challenges involved in constructing it (e.g. he has often been confused with the dedicatee of the first three books of Artemidorus’ (fl. 3rd c. AD) *Onirocritica*, Cassius Maximus), see Michael Trapp , *Maximus of Tyre. The Philosophical Orations* . Translated, with an Introduction and Notes (Oxford: Clarendon Press, 1997), xi–xii and Jeroen Lauwers , Philosophy, Rhetoric and Sophistry in the High Roman Empire: Maximus of Tyre and Twelve Other Intellectuals (Leiden: Brill, 2015), 1–3 . Compare Juan López Cruces and Javier Campos Daroca (eds), Máximo de Tiro: Disertaciones Filosóficas (Madrid: Editorial Gredos, 2005), 7–13 and Brigitte Pérez-Jean and Frédéric Fauquier (eds), *Choix de conferences: religion et philosophie/ Maxime de Tyr* (Paris: Les Belles Lettres, 2014), 13–15.

2 Homer (ca. 750 BC), Plato (428/427 BC–348/347 AD), other Greek philosophers and the classical historiographers loom large. See Michael Trapp, ‘Philosophical Sermons: The “Dialexeis” of Maximus of Tyre’, *Aufstieg und Niedergang der römischen Welt*, 2, 34, 3 (1997), 1945–76, at 1967–70. Maximus’ use of Homer and Plato has been explored by Panagiota Daouti, ‘Homère et Platon chez Maxime de Tyr’ (unpublished PhD thesis: Université Paul Valéry–Montpellier et Université Capodistria d’Athènes, 2015). See also Ryan C. Fowler , Imperial Plato: Albinus, Maximus, Apuleius: Text and Translation, with an Introduction and Commentary (Las Vegas: Parmenides Publishing, 2016), Jan Fredrik Kindstrand , Homer in der zweiten Sophistik (Uppsala: KaWe Composer, 1973) and Heinrich Kämmel, ‘Maximus der Tyrier. Ein Beitrag zur Geschichte der Peadagogik’, *Jahrbücher für Philologie und Pädagogik*, 104 (1871), 1–17.

3 The *Orations* also deal with culture, theology, physics, psychology and epistemology, but never with logic.

4
*Oration* 1.8. Texts follow Michael Trapp’s Teubner edition, *Maximus Tyrius, Dissertationes* (Bibliotheca scriptorum Graecorum et Romanorum Teubneriana) (Stuttgart and Leipzig: in aedibus B.G. Teubneri, 1994). Translations are taken from Trapp, *op. cit.* (note 1) with minor alterations.

5 For an analysis of Maximus’ first oration in the light of his pedagogical rhetoric and philosophical self-presentation, see Jeroen Lauwers , ‘The Rhetoric of Pedagogical Narcissism: Philosophy, Philotimia and Self-Display in Maximus of Tyre’s First Oration’, Classical Quarterly, 59, 2 (2009), 593–607 . Compare Marian Szarmach , Maximos von Tyros. Eine literarische Monographie (Torun: Uniwersytet Mikołaja Kopernika, 1985), 21–4 and Hans Hobein , ‘Zweck und Bedeutung der ersten Rede des Maximus Tyrius’, in F. Leo (ed.), Charites: Friedrich Leo zum sechzigsten Geburtstag dargebracht (Berlin: Weidmannsche Buchh, 1911), 188–219 .

6 See Roger Brock, *Greek Political Imagery from Homer to Aristotle* (New York: Bloomsbury Academic, 2013), xv–xvi.

7 Brief treatments are found in Trapp, *op. cit.* (note 2), 1966–7 and Trapp, *op. cit.* (note 1), xxxix–xl, both drawing on chapter 6 of Michael Trapp’s DPhil thesis, ‘Studies in Maximus of Tyre: A Second-Century Philosophical Orator and his Nachleben, AD 200-1850’ (unpublished DPhil thesis: University of Oxford, 1986).

8 Next to that of steersmanship. The treatment of steersmanship in Maximus seems more monolithic compared to that of medicine because the latter, being a more sophisticated science, allowed the Tyrian to work with more flexibility and hence develop a wider spectrum of elements that were of interest to his philosophical exposition. Generalship and athletics are also often used in imagery in Maximus. An overview of the variety of images found in Maximus is given by Karl Meiser, *Studien zu Maximos Tyrios* (Munich, 1909), 13–24 and Trapp, *op. cit.* (note 7), 156–227. On Maximus and medicine, see Katharina Luchner, *Philiatroi: Studien zum Thema der Krankheit in der griechischen Literatur der Kaiserzeit* (Göttingen: Vandenhoeck & Ruprecht, 2004), 127–38.

9 Maximus has been criticised, *inter alia*, for the meandering and sloppy structure of his thought and the lack of thorough philosophical discussion over a preference for no-frills reference or allusion. For example, Richard Fletcher , Apuleius’ Platonism: The Impersonation of Philosophy (Cambridge: Cambridge University Press, 2017), 41 talks of Maximus as a ‘monolithic’ author who employs ‘a limited array of literary experimentation in his writings’. Compare the term ‘second-hand erudition’ attached to Maximus by Gerald Sandy, *The Greek World of Apuleius: Apuleius and the Second Sophistic* (Leiden; New York: Brill, 1997), 94. Lauwers, *op. cit.* (note 1), 3–6 summarises modern scholarship’s assessment of Maximus and provides further references.

10 This is in line with what rhetorical theory suggested in relation to the function of the simile/comparison: for example, *Rhetoric to Herennium* (ca. 86–82 BC) 4.45.59 and 4.47.60, Quintilian (ca. 35–ca. 100), *Institutes of Oratory* 8.3.72.

11 For example, *Oration* 19.2: ‘I will explain it to you by making up a story in the style of Aesop’s fables’. Maximus also employs the Platonic scheme of using a fable as a prelude to the main argument: ‘Let me now dismiss this fable of mine and convert it into a proper, reasoned argument, which as it advances can draw a comparison between the two lives’, *Oration* 36.2. As Trapp, *op. cit.* (note 1), 284 notes, the shift from *mythos* to *logos* has a precedent in *Protagoras* 324d.

12
*Oration* 6.5.

13
*Oration* 13.4.

14
*Oration* 17.2.

15
*Oration* 21.5.

16
*Oration* 27.6.

17 Also *Oration* 1.6, *Oration* 7.4, *Oration* 11.9, *Oration* 11.12, *Oration* 18.2 and *Oration* 30.3.

18 For Maximus’ young target audience, see, for example, Leonidas Koniaris, ‘On Maximus of Tyre: Zetemata I’, *Classical Antiquity*, 1, 1 (1982), 87–121, at 113–14, Trapp, *op. cit.* (note 1), xx–xxii, Lauwers, *op. cit.* (note 5), 606. The obvious credentials of Maximus’ audience are that they come from the echelons of Roman society and are fluent in Greek and the tradition of the Greek classical past. See also Lauwers *op. cit.* (note 1), 139–43 for the interesting suggestion that Maximus’ final audience was an expanded one that included not just his pupils but also social peers, e.g. the fathers that accompanied their sons to Maximus’ lectures or other gathered onlookers.

19 Compare *Oration* 7.2: ‘Are you prepared to compare the body to a people, and the soul to their ruler? Think then, and put the analogy to work’ (θέασαι τοίνυν καὶ παράβαλε τὴν εἰκόνα).

20
*Phaedo* 87b. On the function of εἰκών in Plato, see, for example, Douglas Cairns, ‘The Tripartite Soul as Metaphor’, in P. Destrée and R.G. Edmonds (eds), *Plato and the Power of Images* (Leiden; Boston: Brill, 2017), 219–38 with further bibliography. Compare Alcinous’ νόησις ἡ κατὰ ἀναλογίαν, *Didaskalikos* 10.5.

21 See, for example, Ryan C. Fowler , ‘Variations of Receptions of Plato during the Second Sophistic’, in H. Tarrant , D.A. Layne , D. Baltzly and F. Renaud (eds), Brill’s Companion to the Reception of Plato in Antiquity (Leiden; Boston: Brill, 2018), 223–49, and 241–3 specifically on Maximus’ familiarity with Platonic philosophy. Maximus’ dependence on Plato is also explored by Trapp, *op. cit.* (note 2), 1946–50 and Trapp, *op. cit.* (note 1), xii–xxx.

22

Martha C. Nussbaum , The Therapy of Desire: Theory and Practice in Hellenistic Ethics (Princeton, N.J.: Princeton University Press, 1994), 14 . See also Jackie Pigeaud , La maladie de l’âme. Étude sur la relation de l’âme et du corps dans la tradition médico-philosophique antique (Paris: Belles Lettres, 1981)6085989, Christopher Gill , ‘Philosophical Therapy as Preventive Psychological Medicine’, in W. Harris (ed.), Mental Disorders in the Classical World (Leiden: Brill, 2013), 339–60, at 343–8.

23 For example, *Discourses* 2.23.30–32.

24 For example, fragment 36 in Plutarch, *On the Control of Anger* 453D–E; cf. Gellius, *Attic Nights* 5.1 = Musonius fragment 49, Hense 1905.

25
*On Anger* 1.15.1.

26 For example, *How to Tell a Flatterer from a Friend* 55A–B.

27
*On Frank Criticism*, fragment 39, fragments 65–66.

28
*Oration* 1.2.

29
*Oration* 1.2.

30
*Kairos* denoted medical intervention – whether in the form of normalising diagnostics or cure – at the right time and at the proper occasion and opportunity. For the notion of *kairos* in ancient medicine, see, e.g. Catherine Eskin , ‘Hippocrates, *Kairos*, and Writing in the Sciences’, in P. Sipiora and J.S. Baumlin (eds), Rhetoric and *Kairos*: Essays in History, Theory, and Praxis (Albany, NY: State University of New York Press, 2002), 97–113 .

31 See also *Oration* 7.1. It is worth noting that, in the programmatic first speech where Maximus establishes the significance of moral virtue, he presents the ideal human end as something pre-eminently stable, using the same participle ἑστός (*hestos*, ‘stable’, *Oration* 1.5). This shows that the notions of stability and a lack thereof are central to Maximus’ moralising, thereby offering a further clue as to why he speaks here of instability rather than of sickness.

32 Maximus talks very emphatically of sickness of the soul in one of his two orations dedicated to the cohabitation and close partnership of body and soul, that is in *Oration* 7.2–3. However, he does so in order to contrast somatic disease with psychic disease, only to conclude that the soul is more important than the body. This is a general lesson that Maximus wishes to pass on to his pupils, aligning himself with the Platonic idea that the body is merely the container of the soul on earth. Another point worth bringing out here is that Maximus’ emphasis on philosophy’s role in maintaining and restoring harmony in the passage above is filtered through the notion of moving between extremes that he mentions twice in a single line (ὑπὸ κενώσεως καὶ πλησμονῆς κυκωμένῳ, ‘in the turmoil of evacuation and repletion’ and οἰκονομοῦσα αὐτοῦ τὴν ἔνδειαν καὶ τὸν κόρον, ‘to regulate the indigence and satiety of a body’). This reflects philosophy’s aim of hitting the mean (μεσότης, *mesotes*).

33 The notion of symmetry features elsewhere in the *Orations*: for example, *Oration* 1.8, *Oration* 2.8, *Oration* 3.1, *Oration* 7.1, *Oration* 15.2, *Oration* 17.1, *Oration* 20.4, *Oration* 22.4, *Oration* 22.7, *Oration* 27.3, *Oration* 36.1, *Oration* 37.5 and *Oration* 39.2.

34
*Oration* 10.5, *Oration* 40.3 and *Oration* 41.3. Euripus is used as an illustration of volatility also in *Oration* 5.6 and *Oration* 28.3; its origin is Plato’s *Phaedo* 90c.

35 Epictetus, for instance, states that: ‘Men, the lecture-room of the philosopher is a hospital (ἰατρεῖόν ἐστιν, ἄνδρες, τὸ τοῦ φιλοσόφου σχολεῖον); you ought not to walk out of it in pleasure, but in pain (οὐ δεῖ ἡσθέντας ἐξελθεῖν, ἀλλ’ ἀλγήσαντας). For you are not well when you come; one man has a dislocated shoulder, another an abscess, another a fistula, another a headache’ (ἔρχεσθε γὰρ οὐχ ὑγιεῖς, ἀλλ’ ὁ μὲν ὦμον ἐκβεβληκώς, ὁ δ’ ἀπόστημα ἔχων, ὁ δὲ σύριγγα, ὁ δὲ κεφαλαλγῶν) (*Discourses* 3.23.30). Compare Dio of Prusa (ca. 40–ca. 115 AD), who in his *Discourse* 77/78.43, encourages frankness, however harsh, at all costs, using the model of the physician.

36 The same holds true for Maximus’ use of medical imagery as a metaphor for public speaking: in *Oration* 25.5, Maximus adds the detail of rhetoric’s ability to please ‘to make the treatment a little easier to digest’, according to Jeroen Lauwers, ‘Self-Advertising Meta-Poetics in Maximus of Tyre’s 25th Oration’, *Wiener Studien*, 125 (2012), 75–84, at 81.

37 Maximus often uses *oikonomia* and its cognates with reference either to divine dispensation (*Oration* 8.8, *Oration* 27.8) or art’s ability to regulate (*Oration* 1.8, *Oration* 5.4).

38 Lauwers, *op. cit.* (note 5), 600, n.31.

39 Trapp, *op. cit.* (note 2), 1949.

40 This observation agrees with Jeroen Lauwers’ statement that Maximus uses a ‘type of philosophy which does not aim at a radical and painful healing of the soul, but rather allows a certain amusement to its practitioners’, Lauwers, *op. cit.* (note 36), 84. See also Michael Trapp, ‘Apuleius of Madauros and Maximus of Tyre’, in R.W. Sharples and R. Sorabji (eds), *Greek and Roman Philosophy 100BC–200AD* (London: Institute of Classical Studies, University of London, 2007), 467–82, at 468, who refers to Maximus’ (and Apuleius’) philosophy as ‘responding to the brief of making *philosophia* safe and enjoyable for the cultivated’.

41 Trapp, *op. cit.* (note 40), 467 labels Maximus a ‘philosophical popularizer’. He was also called ‘a performer of Second Sophistic *paideia*’, Fletcher, *op. cit.* (note 9), 148. Jonathan More, ‘“Like a Bird with the Gift of Reason”: Maximus of Tyre on the Freedom of Diogenes the Cynic’, *Akroterion*, 64 (2019), 91*–*105 touches on Maximus’ affiliation with Imperial-period popular philosophy, but more remains to be said on his moralising Strategies and position in the popular philosophical genre and the tradition of practical ethics. This is a topic I explore in Sophia Xenophontos, ‘How to win Converts to Philosophy: Strategies of Attraction and Persuasion in Maximus of Tyre’s *Dialexeis* 1, 3, 12 and 14’, in Α. Mavroudis (ed.), *Ετος ἦλθε περιπλομένων ἐνιαυτῶν ἑκατοστόν. Τιμητικός τόμος για τον Καθηγητή – Ακαδημαϊκό Ν.Χ. Κονομή [Essays in honour of Professor N. Konomis]* (Athens: Academy of Athens), 259*–*82.

42 Compare the general observation: ‘Maximus himself only rarely makes use of technical terms, usually in order to underline his erudition and thus indirectly promote his credibility and authority as a speaker’, Jeroen Lauwers and Geert Roskam , ‘For Love of an Honourable Name. Prototypical Philosophy and Philosophers in Maximus of Tyre’, in G. Roskam , M. De Pourcq and L. van Der Stockt (eds), The Lash of Ambition. Plutarch, Imperial Greek Literature and the Dynamics of Philotimia (Louvain: Peeters: Société des Études Classiques, 2012), 183–206 , at 192.

43 Widespread interest in medicine among educated laypeople seems to have marked all periods of antiquity. For example, Aristotle (384–322 BC), in his *Politics* 1282a, refers to the man who has studied medicine in the context of his general education.

44 Another fixed formula, used in the same way with reference to emotional health, *Oration* 27.9: ‘This is what produces a happy soul and a healthy life and right opinions, organized into a harmonious blend’ (ὑπὸ ἁρμονίας καὶ κράσεως συνταττόμεναι).

45 Disruption of the balance (δυσκρασία, *dyskrasia*) gives rise to illness. For example, Galen (129–ca. 216 AD), *On Mixtures* 2.1, vol.I.572.3-573.1 in Karl Gottlob Kühn (ed.), *Claudii Galeni Opera Omnia*, 20 vols in 22 (Lipsiae: prostat in officina Libraria Car. Cnoblochii: 1821–1833)=39.3–10 Georgius Helmreich (ed.), *Galeni De temperamentis libri III* (Lipsiae: in aedibus B.G. Teubneri, 1909). On Galen’s theory of mixtures, see Philip van der Eijk, ‘Galen on the Assessment of Bodily Mixtures’, in B. Holmes and K.-D. Fischer (eds), *The Frontiers of Ancient Science: Essays in Honor of Heinrich von Staden* (Berlin: De Gruyter, 2015), 675–98.

46
*Oration* 9.3.

47
*Oration* 27.4 and *Oration* 39.2.

48 For example, Empedocles (ca. 494 BC–ca. 434 BC), fragment 31B96 (H. Diels and W. Kranz, eds, *Die Fragmente der Vorsokratiker*, vol. 1, 6th edn., Berlin: Weidmann, 1951, pp. 345–6), fragment 31B98 (H. Diels and W. Kranz, eds, *Die Fragmente der Vorsokratiker*, vol. 1, 6th edn., Berlin: Weidmann, 1951, p. 346). Plato and the Hippocratics thought along the same lines. Some of these accounts also spoke of balance in the humours of the body (blood, phlegm, black bile, yellow bile). Maximus does not refer to bodily humours.

49
*Oration* 27.3: ‘Start by assigning three separate sciences to three separate sorts of raw material, soul and body and stone; in each case the raw material lacks form, and the sciences, bringing to each its proper configuration, clothe stone in orderly shapes so as to give it the appearance of some recognizable object, the body in harmonious blendings of its constituents so as to produce the proportions that constitute health, and the soul in symmetry and responsiveness so as to produce the adornment of Virtue’ (τὸ δὲ σῶμα ἁρμονίαις καὶ κράσεσιν εἰς ὑγιείας μέτρον, τὴν δὲ ψυχὴν συμμετρίαις καὶ εὐκινησίαις εἰς ἀρετῆς κόσμον).

50 Rather than pointing, for example, to Maximus’ ignorance of medical details. This could hardly have been the case, given that the cultured *pepaideumenoi* in the Imperial period were well-versed in medical issues. In light of this, Lauwers, *op. cit.* (note 5), 606 is right in claiming that Maximus ‘lowered his level to make sure that he will be understood by his pupils’.

51 On medical imagery in Plato, see Joel Warren Lidz, ‘Medicine as Metaphor in Plato’, *The Journal of Medicine and Philosophy*, 20 (1995), 527–41 and, succinctly, Brock, *op. cit.* (note 6), 71–2. Compare Aristotle’s use of medical imagery, Werner Jaeger, ‘Aristotle’s Use of Medicine as Model of Method in His Ethics’, *Journal of Hellenic Studies*, 77, 1 (1957), 54–61.

52
*Oration* 10.3.

53 Aetius (fl. 6th c. AD), *Tetrabiblos* 7.27.4; Leo the physician (9th c.?), *Epitome of Medicine* 3.27. On ancient theories of vision, see Veronique Boudon-Millot , ‘Vision and Vision disorders: Galen’s Physiology of Sight’, in M. Horstmanshoff , H. King and C. Zittel (eds), Blood, Sweat and Tears – The Changing Concepts of Physiology from Antiquity into Early Modern Europe (Leiden; Boston: Brill, 2012), 551–67 and Katerina Ierodiakonou, ‘On Galen’s Theory of Vision’, in P. Adamson, R. Hansberger and J. Wilberding (eds), *Philosophical Themes in Galen* (London: Institute of Classical Studies, School of Advanced Study, University of London, 2014), 235–47. Michael Trapp has noted (personal correspondence) that ἀχλύς also ‘picks up on the imagery of the *Phaedo* myth, where the epistemological limitations of sense-bound humans are likened to living in a mist-filled hollow, rather than up in the bright light of day (109a-110a)’.

54
*Oration* 10.4.

55
*Oration* 3.1.

56
*Oration* 3.6.

57 521d–522a: πεινῆν καὶ διψῆν, 522a; cf. *Oration* 32.6: ὁ κάμνων…καὶ διψῶν καὶ λιμώττων, *Oration* 34.6: δίψα…καὶ λιμός.

58 Maximus is fond of using the notion of fever in the *Orations*, for example, *Oration* 7.4, *Oration* 18.4, *Oration* 25.5 and *Oration* 28.2.

59 καὶ γὰρ ἰατρῶν ὁ φιλάνθρωπος λυπηρότατος, καὶ στρατηγῶν ὁ ἀκριβέστατος, καὶ κυβερνητῶν ὁ ἀσφαλέστατος, *Oration* 14.4.

60 καθάπερ δὲ οἱ ἰατροὶ τοῖς κακοσίτοις τῶν καμνόντων τὰ πικρὰ τῶν φαρμάκων ἀναδεύσαντες προσηνεῖ τροφῇ ἀπέκρυψαν τὴν τοῦ ὠφελοῦντος ἀηδίαν, *Oration* 4.6; cf. *Oration* 17.3 and *Oration* 25.5. This is a widely used comparison: for example, Plato, *Laws* 659e–660a, Xenophon (431–354 BC), *Memorabilia* 4.2.17, Dio of Prusa, *Oration* 33, and the best-known instance, Lucretius (ca. 99–55 BC), *On the Nature of Things* 1.935–950. See also pseudo-Plutarch, *On the Education of Children* 13D.

61 Treated also in *Oration* 1.2 above.

62
*Republic* 389b.

63 οὕτω καὶ ἰατρὸς νοσοῦντα ἐξαπατᾷ…καὶ δεινὸν οὐδέν· ἀλλὰ ἤδη καὶ ψεῦδος ὤνησεν ἀνθρώπους καὶ τἀληθὲς ἔβλαψεν, *Oration* 13.3.

64
*Oration* 17.3.

65
*Republic* 398a, 472d–e. Karl Dürr, ‘Sprachliche Untersuchungen zu den Dialexeis des Maximus von Tyrus’, *Philologus,* Suppl.b 8 (1899–1901), 1–156, at 83–6 lists Maximus’ linguistic borrowings from Platonic dialogues.

66
*Oration* 39.2.

67 Perhaps pleasurable foods; see Olympiodorus’ (495–570 AD), *Commentary on the Gorgias* 32.15. Alternatively, this may be a case of mechanical employment of the stock antithesis of the useful and the pleasure of poetry applied straightforwardly and not so accurately to medicine. Or Maximus’ intended meaning could also be that medicine produces the pleasure of a healthy state. I owe these two interpretations to Michael Trapp (personal correspondence).

68
*Oration* 25.5.

69 Elsewhere he presents drug sellers as mimicking physicians, comparing them to sophists who mimic philosophers, *Oration* 20.3.

70
*Oration* 17.; cf. *Oration* 21.5.

71
*Oration* 14.8.

72 An enduring one, as seen above, *Oration* 17.3, *Oration* 25.5.

73 For example, *Oration* 6.4, *Oration* 17.3, *Oration* 40.3. Unlike, for example, Hippocrates who is referred to only twice, and then very briefly (e.g. *Oration* 17.3, *Oration* 22.7).

74 In the *Symposium* 186e, Asclepius is said to be the inventor and governor of the medical art.

75 Compare above all, Aelius Aristides’ *Sacred Tales.*

76
*Oration* 21.5.

77
*Oration* 21.5: ‘I take it that it is impossible for the body to be nourished without food being administered, and the teeth chewing it, and the intestines receiving it, and the whole digestive system doing its work as the nourishment is distributed to the body’. The description of the intermediaries that ensures a coherent bodily composition also sounds commonsensical: ‘Hair and nails are softer than bone, slenderer than muscle, drier than blood, and tougher than flesh’, *Oration* 9.4.

78
*Oration* 20.3.

79
*Oration* 39.2. See Dürr, *op. cit.* (note 75), 14–15.

80
*Oration* 9.7. On Maximus’ demonology compared to that of Apuleius, see Trapp, *op. cit.* (note 40), 472–81, Geoffrey C. Benson, ‘Seeing Demons: Autopsy in Maximus of Tyre’s Oration 9 and its Absence in Apuleius’ on the God of Socrates’, *Ramus*, 45, 1 (2016), 102–31, and Geert Roskam, ‘Socrates’ daimonion in Maximus of Tyre, Apuleius, and Plutarch’, in F. Frazier and D. Leão (eds), T*yche et Pronoia. La marche du monde selon Plutarque* (Coimbra: Classica Digitalia Universitatis Conimbrigensis: Centre de Estudos Clássicos e Humanísticos da Universidade de Coimbra, 2010), 93–108.

81
*Oration* 9.7. Trapp, *op. cit.* (note 1), 83, n.24, notes that this was ‘a special favour, also enjoyed by Antiochus of Aegae (Philostr. *Vit. Soph.* 2. 568) and Proclus (Marinus *Vit. Prod.* pp. 79-80)’. For the popularity of Asclepius and his cult in this period, see Ido Israelowich , Patients and Healers in the High Empire (Baltimore: Johns Hopkins University Press, 2015).

82 Credibility: Dürr, *op. cit.* (note 75), 4; memorable: Benson, *op. cit.* (note 92), 108 and 113.

83
*Oration* 6.2.

84
*Oration* 4.1–3.

85
*Oration* 28.1.

86
*Oration* 28.1–4.

87 Maximus must have derived the association of Chiron with medicine from *Iliad* 4.219, Pindar (ca. 518–ca. 438 BC), *Pythian* 3.1–8 and popular culture because in Plato, Chiron appears twice (*Republic* 391c, *Hippias Minor* 371d), but only as Achilles’ pedagogue. Maximus twice links Chiron to Asclepius in the *Orations*, *Oration* 36.5, *Oration* 40.3.

88 For example, *Oration* 9.4; cf. *Oration* 2.3.

89 This is a ubiquitous theme is Galen’s *Recognising the Best Physician* and *On Prognosis.* See Sophia Xenophontos, *Medicine and Practical Ethics in Galen* (Cambridge: Cambridge University Press, forthcoming), chs. 7 and 8.

90
*Republic* 405c ff.

91
*Oration* 4.1–3.

92
*Oration* 5.4.

93 For example, Epictetus’ *Discourses* 2.5.25 (the foot as part of the body), Seneca’s *On Providence* 3.2 (amputation saves the body) or Marcus Aurelius’ (121–180 AD) *Meditations* 5.2 (our misfortunes are ‘bitter pills’ which we should welcome in the hope of health). Maximus uses the same idea in *Oration* 32.10.

94 God is depicted as a physician also in Plutarch’s *On Slowness to Punish* 549F–550A. Plutarch’s image refers to the way God administers punishment as a medicine. Maximus’ God-doctor is caring, not an avenger.

95 Just like his relation to Stoic accounts of divine providence in *Orations* 13 and 41. See Trapp, *op. cit.* (note 1), xxviii–xxx and 322.

96
*Oration* 12.8. Maximus also couples medicine with justice in an interesting passage where he says that doctors ‘if they are just and love their fellow men, would pray for their art to die out along with the diseases it exists to treat’, *Oration* 24.2.

97 For example, Wendy Mayer , ‘Medicine and Metaphor in Late Antiquity: How Some Recent Shifts are Changing the Field’, Studies in Late Antiquity 2, 4 (2018), 440–63.

98
*Oration* 13.4.

99
*Oration* 8.7.

